# A recombinant pseudotyped lentivirus expressing the envelope glycoprotein of Hantaan virus induced protective immunity in mice

**DOI:** 10.1186/1743-422X-10-301

**Published:** 2013-10-05

**Authors:** Lan Yu, Wentao Bai, Xingan Wu, Liang Zhang, Lei Zhang, Puyuan Li, Fang Wang, Ziyu Liu, Fanglin Zhang, Zhikai Xu

**Affiliations:** 1 Department of Microbiology, Fourth Military Medical University, Xi’an, 710032, China; 2 Department of Minimally Invasive Surgery of Military General Surgery Center, General Hospital of People’s Liberation Army Chengdu Military Region, Chengdu 610083, China

**Keywords:** Recombinant pseudotyped lentivirus, Hantaan virus, Glycoprotein, Protective immunity

## Abstract

**Background:**

Hantaviruses cause acute hemorrhagic fever with renal syndrome (HFRS). Currently, several types of inactivated HFRS vaccines are widely used, however the limited ability of these immunogen to elicit neutralizing antibodies restricts vaccine efficacy. Development of an effective vaccine to overcome this weakness is must.

**Methods:**

In the present study, a recombinant pseudotyped lentivirus bearing the hantaan virus (HTNV) envelope glycoproteins (GP), rLV-M, was constructed. C57BL/6 mice were immunized with the rLV-M and a series of immunological assays were conducted to determine the immunogenicity of the recombinant pseudotyped lentivirus. The humoral and cell-mediated immune responses induced by rLV-M were compared with those of the inactivated HFRS vaccine.

**Results:**

Indirect immunofluorescence assay (IFA) showed the rLV-M expressed target proteins in HEK-293cells. In mice, the rLV-M efficiently induced GP-specific humoral responses and protection against HTNV infection. Furthermore, the rLV-M induced higher neutralizing antibody titers than the inactivated HFRS vaccine control.

**Conclusions:**

The results indicated the potential of using a pseudotyped lentivirus as a delivery vector for a hantavirus vaccine immunogen.

## Introduction

Hantaviruses constitute a genus of the family Bunyaviridae. Hantaviruses are spherical, enveloped viruses with a genome consisting of three segments of single-stranded, negative-sense RNA. The three segments are designated as the small (S), medium (M) and large (L) segments, which encode the nucleocapsid protein (NP), the precursor to the virion envelope glycoproteins (GP), and RNA polymerase, respectively [1]. The glycoprotein precursor is co-translationally cleaved to generate the glycoproteins Gn and Gc, which form heterodimers in the endoplasmic reticulum [2].

Hantavirus infections are known to cause two serious and often fatal human diseases, hemorrhagic fever with renal syndrome (HFRS) and hantavirus pulmonary syndrome (HPS) [3]. Four hantavirus species are responsible for most cases of HFRS in Asia and Europe: Hantaan virus (HTNV), Seoul virus (SEOV), Puumala virus (PUUV) and Dobrava virus (DOBV) [4]. Most HPS cases in North America are caused by Sin Nombre virus (SNV) or in South America, Andes virus (ANDV) [5,6]. Although vaccination, along with public education and rodent control measures, have coincided with a reduction in HFRS, now totaling less than 20,000 cases per year, China still has the highest number of HFRS cases and deaths in the world [7]. The majority of Chinese cases are caused by HTNV and SEOV.

Cultured cell-derived inactivated vaccines against HTNV and SEOV were developed in China, North Korea and Korea [8]. These vaccines induce strong humoral immune responses, but neutralizing antibodies are difficult to elicit. [9]. Researches have hypothesized that glycoproteins play an important role in eliciting neutralizing antibodies capable of protecting humans and animals from Hantavirus infection [10]. Several lines of evidence support this assumption. Firstly, monoclonal antibodies (mAbs) directed against Gn and Gc, but not NP, display neutralizing activity in vitro [11]. Secondly, passive immunization with HTNV envelope glycoprotein-directed mAbs protects suckling mice from a lethal dose of virus [12]. Thirdly, vaccination of animals with glycoproteins of HTNV elicits neutralizing antibodies and protects rodents against infection with HTNV [13,14]. Thus, Hantavirus glycoproteins are considered protective antigens, and are the main immunogen candidates for genetically engineered vaccines targeting for hantaviruses.

Lentiviral vectors (LVs) have been shown to be excellent delivery vehicles for antigens of infectious diseases or cancers for vaccination purposes, capable of eliciting effective cellular immunity and humoral responses [15]. LVs are capable of delivering large antigens to aid the induction of antigen-specific immunity from antigen-presenting cells (APCs). Another key feature of LVs is the low anti-vector immunity inherent in host organisms, allowing LVs and transgene-expressing cells to avoid rapid clearance. This results in efficient antigen expression and presentation in vivo and allows for the possibility of multiple rounds of immunizations [16]. It has been previously shown that the direct injection of antigen-expressing LVs has been very effective in generating neutralizing antibodies against WNV [17].

In this study, we constructed a recombinant pseudotyped lentivirus, rLV-M, expressing the HTNV GP. C57BL/6 mice immunized with rLV-M developed HTNV-specific cellular and humoral immune responses. Neutralizing antibodies were produced at high titers and protected mice from infection with HTNV to a certain extent. We believe that rLV-M demonstrates the potential of using a pseudotyped lentivirus as a delivery vector for a hantavirus vaccine immunogen.

## Results

### Expression of HTNV Gn and Gc by recombinant pseudotyped lentivirus

**Figure 1:**
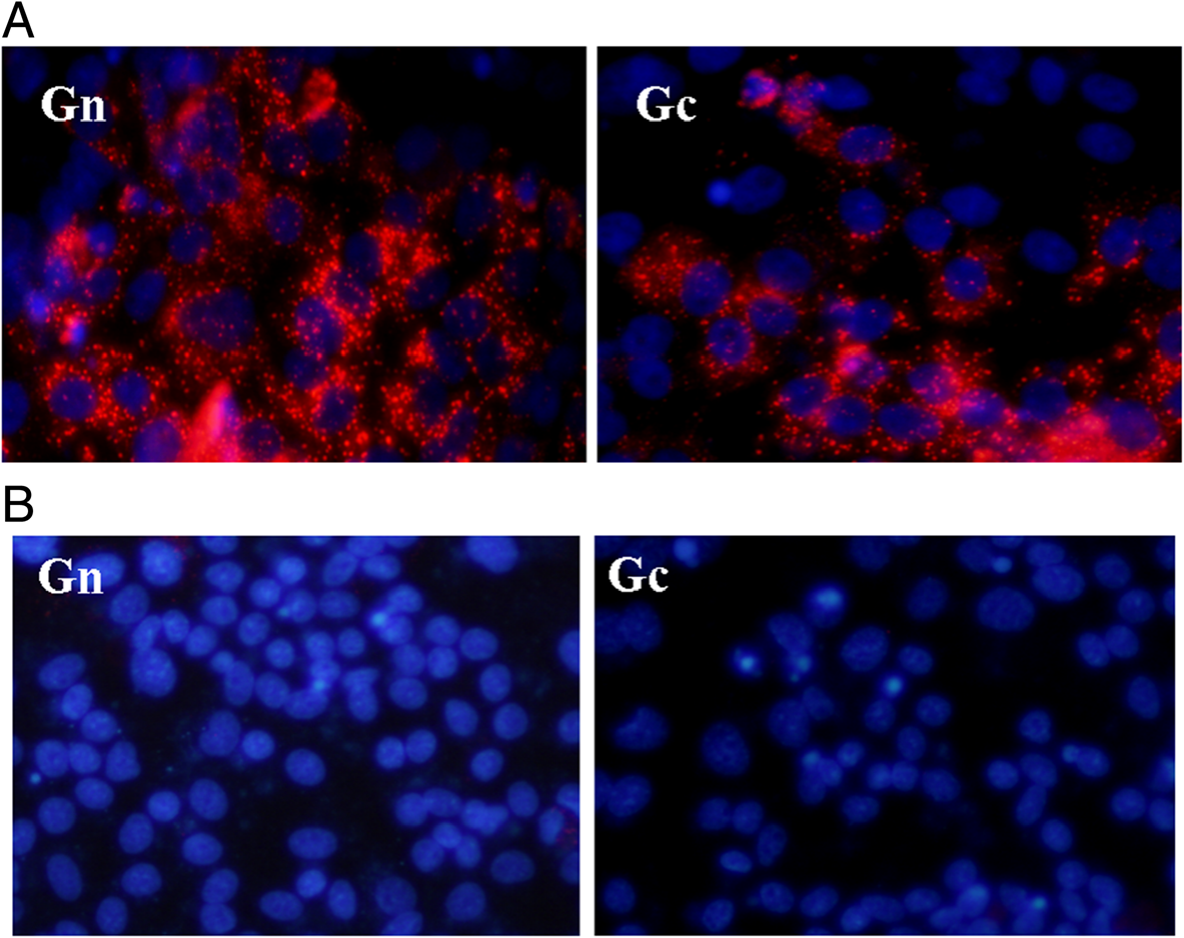
**Immunofluorescence detection of HTNV Gn and Gc expression in HEK 293 cells infected by rLV-M.** Infected HEK 293 cells were treated with mouse monoclonal antibody 6F7 or LV48A to detect Gn or Gc respectively. TRITC-conjugated goat anti-mouse antibody was used demonstrate the positive fluorescence signals. Normal HEK 293 cells were used as negative control. **(A)** rLV- M-infected HEK 293 cells; **(B)** normal HEK 293 cells.

HEK293 cells were infected with the recombinant pseudotyped lentivirus rLV-M expressing HTNV GP. After 48h expression of both Gn and Gc was detected by IFA (Figure 1).

### Titration of recombinant pseudotyped lentivirus containing HTNV glycoproteins

HEK293 cells were infected with rLV-M, and GFP-expressing cells were detected by fluorescence microscope 48 h after infection. The 10^-4^ dilution was used to calculate the titer since the number of GFP-positive cells fell into the desired range of 1–10. The number of GFP-positive cells was four, calculated as follows: 4×10^4+3^ TU/ml. Thus the titer of rLV-M was 4×10^7^ TU/ml.

### Induction of humoral immune responses by rLV-M immunization

**Figure 2:**
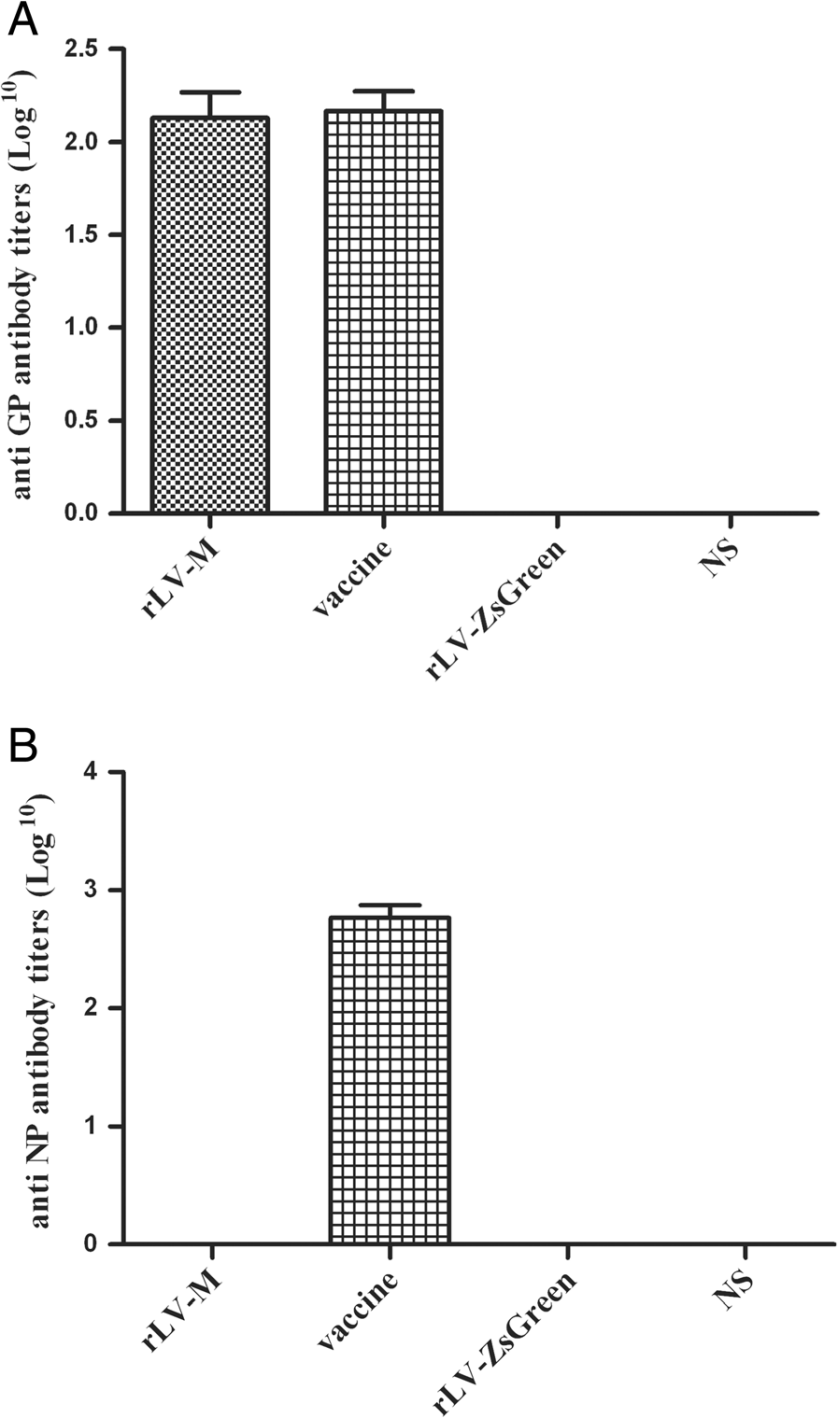
**HTNV GP- and NP-specific antibody titer in the serum of immunized mice (n=8).** Anti-GP and NP antibodies were measured using ELISA. **(A)** HTNV GP specific antibody. **(B)** HTNV NP specific antibody. Similar levels of anti-GP antibodies were detected in mice immunized with rLV-M and HFRS inactivated vaccine. NP-specific antibodies were only induced in mice inoculated with the HFRS inactivated vaccine.

In all mice immunized with rLV-M or HFRS inactivated vaccine, similar levels of anti-GP antibodies were detected after the third immunization (Figure 2A) and the geometric mean titers (GMT) were 1:134.543 and 1:146.721, respectively. NP-specific antibodies were only detected in the mice inoculated with HFRS inactivated vaccine (Figure 2B). Neither GP-specific antibody nor NP-specific antibody was detected in the rLV- ZsGreen or NS group. These results indicate that rLV-M was able to induce GP-specific antibodies in mice.

Neutralizing antibody titers were detected by the cell microculture neutralization test. As shown in Table 1, all eight mice immunized with rLV-M developed neutralizing antibodies after the third immunization, and the neutralizing titers ranged from 1:80 to 1:160, which were much higher than those induced by the HFRS vaccine (p<0.05). Neutralizing antibodies were not detected in mice immunized with rLV-ZsGreen and NS. The results suggest that immunization with rLV-M elicits neutralizing antibodies more efficiently than the inactivated vaccine.

**Table 1:** Neutralizing antibody titer detection in the serum of immunized mice (n=8)

**Mouse**	**rLV-M**	**rLV-ZsGreen**	**HFRS vaccine**	**NS**
1	1:80	-	1:20	-
2	1:80	-	1:10	-
3	1:160	-	1:20	-
4	1:80	-	1:20	-
5	1:160	-	1:10	-
6	1:80	-	1:20	-
7	1:160	-	1:20	-
8	1:80	-	1:20	-

### Frequency of IFN-γ, IL-2, IL-10 and IL-4-secreting cells in splenocytes after immunization with rLV-M

**Figure 3:**
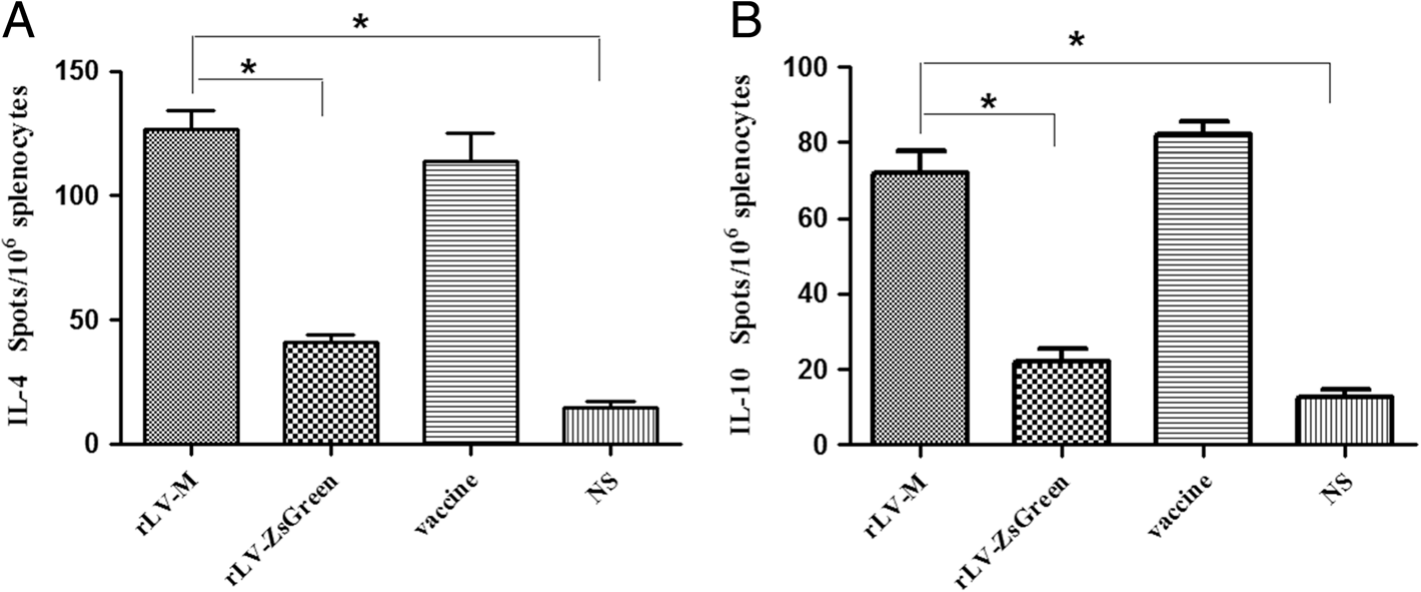
**Frequency of antigen-specific IL-10- or IL-4-secreting T cells in splenocytes of immunized mice (n=4).** Number of GPspecific IL-4-secreting T cells **(A)** and IL-10-secreting T cells **(B)** were evaluated using ELISPOT assays. Results are shown as the mean value of the number of spots observed for 10^6^ spleen cells, obtained from triplicate wells. The rLV-M induced effective IL-10 and IL-4 responses, which were higher than the rLV-ZsGreen control and NS group (*p<0.05), and equivalent to the inactivated vaccine group.

**Figure 4:**
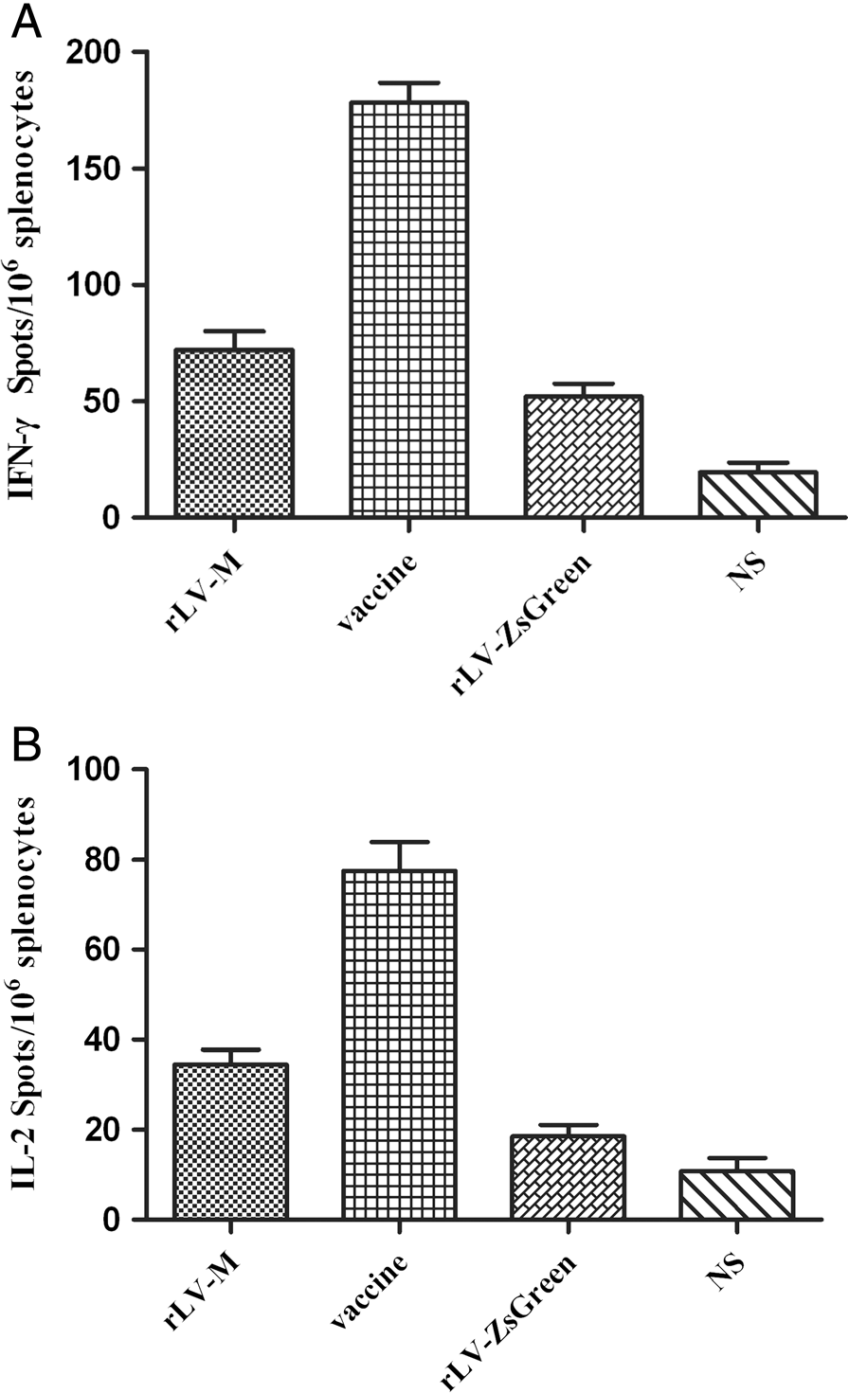
**Frequency of antigen-specific IFN-γ- or IL-2-secreting T cells in splenocytes of mice immunized (n=4).** Number of GP specific IFN-γ-secreting T cells **(A)** and IL-2-secreting T cells **(B)** were evaluated using ELISPOT assays. Results are shown as the mean value of the number of spots observed for 10^6^ spleen cells, obtained from triplicate wells. The IFN-γ and IL-2 levels did not change significantly in the rLV-M group.

The frequencies of IFN-γ, IL-2, IL-4 and IL-10-secreting cells in splenocytes of immunized mice were determined by the ELISPOT assay. Immunization with rLV-M induced a significantly higher frequency of GP specific IL-4 and IL-10-secreting cells than immunization with rLV-ZsGreen control and NS (Figure 3), and an equivalent level to the inactivated vaccine group. However, the IFN-γ and IL-2 levels did not change significantly for the rLV-M immunization group (Figure 4).

### Evaluation of the protective efficacy of rLV-M in mice

**Figure 5:**
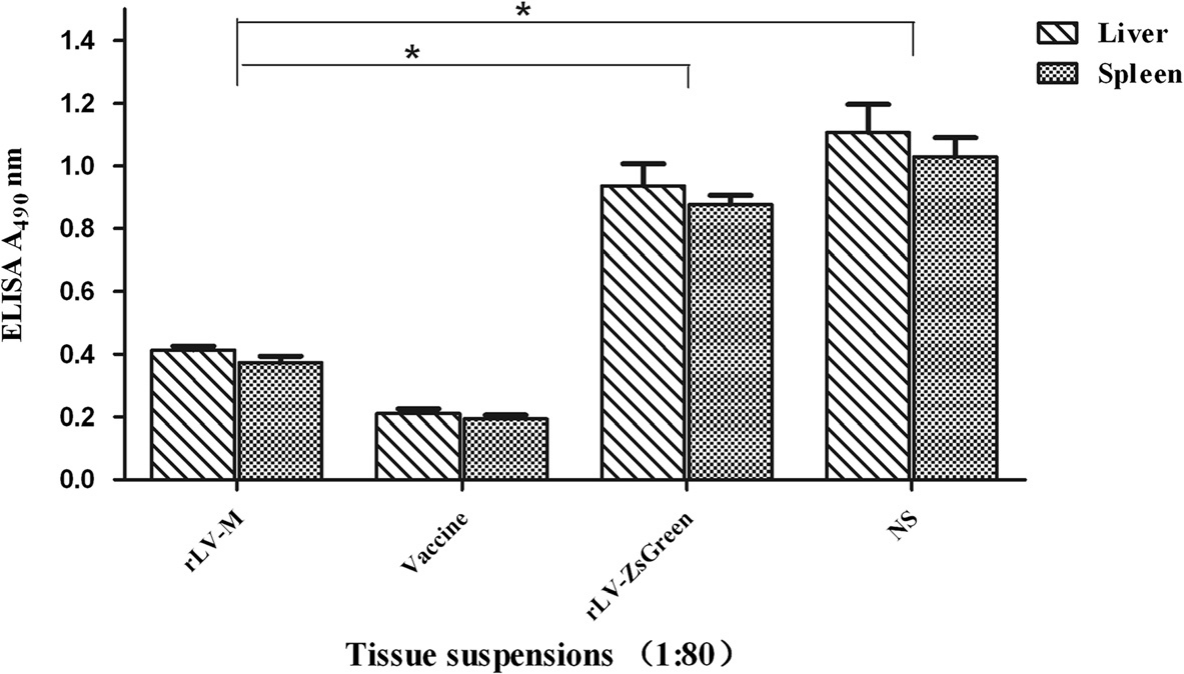
**Detection of HTNV antigen in the livers and spleens of mice (n=4).** HTNV antigen was detected by ELISA. Mice tissue suspensions were diluted 1:80. Data is presented as the mean of A_490_. The level of HTNV antigens in the rLV-M immunized mice were higher than the inactivated vaccine group but significantly lower than the rLV-ZsGreen and NS control groups (*p<0.05).

To assess the protective immunity induced by rLV-M, mice were challenged with HTNV ten days after the final booster immunization. According the result of the study made in our laboratory before, we found that viral antigens could be detected from the liver and spleen samples early after infection in the C57BL/6 mice [18]. So we measured the HTNV specific antigen present in the livers and spleens by ELISA after 3 days. As shown in Figure 5, although the level of antigen found in the rLV-M immunized mice were higher than the inactivated vaccine group but significantly lower than the rLV-ZsGreen and NS control groups (p<0.05). This suggested that the rLV-M induced immunity could limit HTNV infection.

## Discussion

Vaccination is the most cost-effective means for preventing the spread of viral infectious diseases. In the case of HFRS, great effort has been spent on the development of effective vaccines. Several types of inactivated vaccines for HFRS have been licensed in china [19] however inactivated vaccines are poorly immunogenic and elicit few neutralizing antibodies [20]. Increasing an emphasis is being placed on developing genetically engineered vaccines. Glycoproteins have become a major candidate for the HFRS genetically engineered vaccine immunogen. Previous studies have found that DNA vaccines that express the Gn and Gc glycoproteins of hantaviruses can elicit neutralizing antibodies against these proteins and can confer protection against infection in Syrian hamsters [21-23]. Similarly, vaccination with Gn/Gc protein expressed in insect cells (baculovirus recombinant virus system) elicited neutralizing antibodies and protected hamsters from infection with HTNV [12]. In both the vaccinia and baculovirus systems, vaccination with Gn/Gc provided more complete protection than Gn or Gc alone [12]. Neutralizing antibody responses to Gn/Gc in the aforementioned vaccine studies correlated with protection, suggesting that neutralizing antibodies play an important role in preventing hantavirus infection. Passive transfer of neutralizing monoclonal antibodies specific to either Gn or Gc protected hamsters against HTNV infection [12,24], supporting the idea that neutralizing antibodies alone can confer protection.

This is not the first pseudotype preparation for hantavirus vaccine candidate. It has been reported that a VSV pseudotype including HTNV Gn and Gc (VSV-G*HTN) was constructed to study the antigenicity of HTNV glycoproteins. The VSV system has the ability to grow to high titers in a variety of cell lines and allows the generation of recombinant viruses that express foreign proteins [25,26] In this study, an HTNV pseudotyped lentiviral vector carrying HTNV Gn and Gc was developed and the lentiviral vector system also has its own advantages. The HIV-derived lentiviral packaging system we used in this study was named ‘third-generation self-inactivating LV’. The features attributed to this designation include (i) Tat and the four accessory genes of HIV were deleted from the viral packaging system, consisting of four plasmids used to transfect the 293T packaging cell line [27]; and (ii) inclusion of a 400-nucleotide deletion in the 3’long terminal repeat (LTR), which is copied to the 5’LTR upon reverse transcription, thereby abolishing the 5’LTR promoter activity and reducing the risk of vector mobilization with the wild-type virus [28]. Since the original lentivirus envelope protein (gp120) restricts infection to only a subset of T cells, is unstable, and complicates production of LVs, vectors are pseudotyped with vesicular stomatitis virus glycoprotein (VSV-G). LVs have been validated as highly efficient and robust mediators for gene transfer in a wide variety of in vitro and in vivo settings [29,30]. Furthermore, such lentiviral vectors represent powerful and safe gene transfer tools since they can be easily produced at high titers and the generation of replication-competent retroviruses is very unlikely [14]. These advantages make the lentiviral vector system an excellent choice.

In this study, the pseudotyped lentivirus rLV-M was successfully applied as an HTNV vaccine for the induction of protective immunity in mice. We found that the rLV-M was able to induce higher titers of neutralizing antibody than the HFRS inactivated vaccine, and conferred protective immunity for hantavirus challenge in the mouse model. A recombinant retrovirus-based pseudotype including hepatitis C virus (HCV) glycoproteins has been reported to induce HCV-specific cellular and neutralizing immune responses in mice [31]. Similarly, a VSV pseudotype including HTNV Gn and Gc (VSV-G*HTN) was able to induce neutralizing antibodies and confer protective immunity for hantavirus challenge in the mouse model [32]. The results of these reports confirm that recombinant pseudotyped virus can induce effective immune responses than inactivated virus, probably because they display viral envelope glycoproteins in a native conformation.

GP and NP are both the major structural proteins of HTNV. NP is most immunogenic and can elicit high titer and a long-lasting antibody responses. Furthermore, NP contains antigenic sites associated with the cytotoxic T-lymphocyte (CTL) response [33] and can induce a significant cellular immune response [34,35]. GP is presumed to be the major target neutralizing antibodies during Hantavirus infection, and plays an important role in eliciting protective humoral immune responses. However, GP immunogenicity is weak, the antibodies elicited by GP are produced later, and the titer is low [11,36]. We found that recombinant pseudotyped lentivirus rLV-M could stimulate murine spleen cells to secrete IL-4 and IL-10 in a higher level, whilst the levels of secreted INF-γ and IL-2 did not differ significantly from the rLV-ZsGreen control group. This suggests that the protective efficacy elicited by rLV-M was primarily dependent on the humoral immune response.

Neutralizing antibodies can bind extracellular free virus and eliminate the ability of virions to infect target cells. Neutralizing antibodies are presumed to play an important role in clearance of free virus, and to be the major mediators of the protective humoral immune response. One of the main indexes of antiviral vaccination (especially acute viral infection) effectiveness is its ability to induce neutralizing antibodies, especially high titers of neutralizing antibodies. Lentiviral vectors can infect dividing and non-dividing cells, accommodate large fragments of exogenous gene, express target genes for extended periods, and are not inherently immunogenic [16]. Iglesias et al. [17] constructed a lentiviral vector-based vaccine containing West Nile virus (WNV) envelope glycoprotein and successfully induced a specific humoral response and protection against WNV infection in a mouse model of WNV encephalitis. In this study, the HFRS inactivated vaccine was used as one of the controls. In comparison to the inactivated vaccine immunized group, mice immunized with rLV-M induce the same titer of specific antibodies and higher titer of neutralizing antibodies. The results highlight the potential of using the pseudotyped lentivirus as a tool for developing a vaccine that may induce neutralizing antibody more efficiently.

Because there are no disease models for hantavirus in adult rodents, we evaluated whether our recombinant pseudotyped lentivirus could protect against infection rather than prevent disease. After infection with the HTNV 76–118 strain, the HTNV specific antigen in the livers and spleens of mice was detected by ELISA [18]. Although the level of antigens in the rLV-M immunized mice were higher than the inactivated vaccine group, they were significantly lower than the rLV-ZsGreen and NS control groups. This suggested that neutralizing antibodies in rLV-M immunized mice can effectively limit the infection. We found that the immune protective effect induced by rLV-M was weaker than the inactivated vaccine group, probably because that inactivated vaccine simultaneously induced humoral and cellular immune responses whilst rLV-M only induced humoral responses.

Clearly more studies are needed to explore the potential of the pseudotyped lentivirus system in development of a genetically engineered vaccine for HFRS. However we have demonstrated that this system can induce a strong neutralizing antibody response to HTNV glycoproteins.

## Materials and methods

### Cells, virus and antibodies

The human embryonic kidney cell line 293 (HEK 293, ATCC, Rockville, MD, CRL 11268) and 293T cells (ATCC, Rockville, MD, CRL 1573) were maintained in Dulbecco modified Eagle medium (DMEM, Gibco, Grand Island, USA) supplemented with 10% fetal bovine serum (FBS, HyClone, Logan, UT). The E6 clone of Vero cells (ATCC C1008; Cell Repository line 1586) were maintained in RPMI-1640 (Invitrogen, Carlsbad, USA) supplemented with 10% fetal calf serum (FCS, Gibco, Grand Island, USA). All cells were incubated at 37°C in 5% CO_2_. The HFRS inactivated vaccine (YOUERJIAN®) was purchased from Zhejiang Tianyuan Bio-Pharmaceutical Co. Ltd. (Zhejiang, China).

Monoclonal antibodies (mAb) 1A8 (specific to the HTNV NP), 3G1 (with a high neutralizing activity against HTNV) were prepared in our laboratory [37]. The mAb 6F7 (specific to the HTNV Gn protein) and LV48A (specific to the HTNV Gc protein) were kindly provided by Hang C.S. (Chinese Center for Disease Control and Prevention) [38]. Sp2/0 ascites and HTNV 76–118 strain were provided by our laboratory. Purified GP and NP of HTNV were purchased from Lanzhou Biological Product Academy (Lanzhou, China).

### Animals

Six- to eight-week-old female C57BL/6 mice were purchased from the animal research center of the 4th Military Medical University (Xi'an, China). Mice were housed in isolated and ventilated cages, and all animal research was conducted in accordance with procedures described in the Guide for the Care and Use of Laboratory Animals (NIH Publications No. 80–23, revised 1978). The animal work was approved by the Fourth Military Medical University Medical Ethics Committee. The approval number was XJYYLL-2012564.

### Construction of lentiviral expression vector and recombinant pseudotyped lentivirus

The HTNV glycoprotein precursor coding region (M) was designed according to previously cloned cDNA of isolate 76–118 (GeneBank accession number: Y00386) [39] and synthesized in the pMD19-T simple vector with the desired restriction enzyme sites by the Takara company. The vector was excised using EcoRI and BamHI and subcloned into the lentiviral expression vector pLVX-mCMV-ZsGreen1 (Biowit Technologies Ltd, China). ZsGreen1 is a human codon-optimized variant of the reef coral Zoanthus sp. green fluorescent protein (GFP), ZsGreen. The vector expresses the proteins from a bicistronic mRNA transcript, allowing ZsGreen1 to be used as an indicator and marker. The reconstructed vectors were named pLVX-M. Packaging plasmids pGag/Pol (coding for HIV-1 Gag-Pol), pRev (coding for HIV-1 Rev) and pVSV-G (coding for the G protein of VSV), were purchased from Biowit Technologies Ltd.

80% confluent 293T cell monolayers in 10cm plates were co-transfected with pLVX-M and the packaging vectors using HET transfection reagents (Biowit Technologies Ltd, China), as recommended by the manufacturer and incubated for 12 h at 37°C in 5% CO_2_. After removing the flask and adding 10 ml of fresh medium, the cells were incubated for 24h. The culture supernatant was clarified by low-speed centrifugation and stored at −80°C. The recombinant pseudotyped lentivirus was designated rLV-M. The empty vector control lentivirus, rLV- ZsGreen, was prepared in the same way.

### Titration of recombinant pseudotyped lentivirus

The viral titers were determined using the Lentiviral Rapid Titer Kit (Biowit Technologies Ltd, China) as recommended by the manufacturer. After infection with 10μl of serially diluted virus stock, the fluorescent GFP-expressing HEK293 cells were counted under a fluorescence microscope (Olympus, Japan). The following formula is used to calculate the titer: N×10^m+3^TU/ml (N denotes the number of GFP-positive cells and 10^m^ the highest dilution).

### Immunofluorescence staining of the protein Gn and Gc expressed by recombinant pseudotyped lentivirus

The expression of Gn and Gc was detected by an indirect immunofluorescent antibody (IFA). HEK293 cells, grown on 24-well plates, were infected with virus stock at a multiplicity of infection (MOI) of 10 TU/cell. After 48h, the HEK293 cells were fixed with methanol for 20 min. Then treated with mouse monoclonal antibody 6F7 against Gn or mouse monoclonal antibody LV48A against Gc for 1 h at 37°C. After washing with PBS, rhodamine (TRITC)-conjugated goat polyclonal antibodies against mouse immunoglobulin G (IgG; Biaworld), diluted 1:200, were added to the well. After 1 h incubation at 37°C, cells were washed and treated with dihydrochloride salt (DAPI; Beyotime) and examined with a fluorescence microscope.

### Immunization of mice

C57BL/6 mice were divided into four groups including one experimental group and three control groups (n = 8 per group). The experimental groups were injected intramuscularly in the hind limb with 100 μl inoculum containing 10^7^ TU of rLV-M per mouse; the control groups were injected with 100 μl normal saline (NS) or 100 μl 10^7^ TU of rLV-ZsGreen or 10 μl HFRS inactivated vaccine per mouse, respectively. The HFRS vaccine was an HFRS bivalent inactivated vaccine for humans, and the immunization dose was calculated according to the ratio of human and mouse body surface area [40]. All immunizations were given three times at 2-week-intervals. Mice sera were collected individually via tail vein puncture at 2, 4 and 6 weeks from the first day of immunization.

### Detection of HTNV-specific antibodies and neutralizing antibody

HTNV NP- and GP-specific antibody titers were determined by an indirect enzyme-linked immunosorbent assay (ELISA). Purified NP or GP was used as the coating antigen. Serial dilutions of sera beginning with 1:10 were added to the plates. Anti-NP mAb 1A8 or HFRS patient serum diluted at 1:100 were used as positive controls. HRP-conjugated anti-mouse or anti-human antibodies were used as the detecting antibodies and OPD was used as the substrate. To make the reaction reacted just enough, the colorimetric reaction was stopped by adding 2 M H_2_SO_4_ about 20 min after added to the plates and the optical density (OD) at 490 nm was determined using a standard ELISA plate reader. The antibody titers were defined as the reciprocal of the serum dilution with the highest positive response.

The cell microculture neutralization test was performed on monolayers of Vero E6 cells grown in a 96-well tissue culture plate with the HTNV 76–118 strain. Cells grown in RPMI-1640 medium supplemented with 10% FCS were plated at a density of 2 × 10^4^ cells per well 18–24 h before testing. The 0.22μm filtered sera were serially diluted twofold from 1:10 in RPMI-1640 containing 2% FCS. The 100 TCID_50_ HTNV was mixed with the diluted sera and incubated at 37°C for 90 min. Then, the mixture was applied to cell monolayers and incubated at 37°C for 90 min. After removing the mixture and adding fresh medium, the cells were incubated for a further 9–11 days. Thereafter, the cells were lysed by three consecutive freeze–thaw cycles. HTNV antigen in the cell lysates was detected by sandwich ELISA [20]. The mAb 1A8 was used as a coating antibody, and HRP-conjugated 1A8 was used as the detecting antibody. The mAb 3G1 and Sp2/0 ascites were used as positive and negative controls, respectively. The absorbance at 490 nm was read with a standard ELISA plate reader. The neutralizing antibody titer was defined as the maximum dilution of serum that inhibited HTNV infection in 50% of the cells.

This cell microculture neutralization test was established by our laboratory and has the same validity in measuring neutralizing antibody titers as the plaque assay [41].

Furthermore, our ELISA-based neutralization test is more convenient in operation and more stable in results [41]. It was also used in other experiments in our laboratory reported before [40,42].

### Detection of cytokines secreted by T cells

To determine the fraction of T cells capable of responding to IFN-γ stimulus, an enzyme-linked immunosorbent (ELISPOT) assay was performed on single-cell suspension of spleen using the murine IFN-γ ELISPOT kits (Mabtech, AB, Sweden). Ten days after the final booster immunization, four immunized mice were sacrificed. The spleens were removed and purified in lymphocyte separation medium (Sigma, USA). A cytokine-specific antibody against mouse IFN-γ was immobilized on a 96-well ELISPOT plate, and incubated overnight at 4°C. Splenocytes were plated in triplicate at 1×10^6^ cells per well in 100μl of media and co-incubated with purified HTNV GP antigen in a 5% CO_2_ incubator for 18 h. Splenocytes stimulated with concanavalin A (ConA, Sigma, USA) were used as a positive control, and splenocytes incubated with 100μl 2% FCS DMEM were used as negative or background controls. After removing the cells, the plates were incubated with biotinylated anti-mouse IFN-γ detection antibody for 2 h at room temperature (RT). After washing, plates were incubated for 1 h with streptavidin-enzyme conjugate at 37°C. Spots were developed with the TMB substrate after 5–15 min incubation at RT. The reaction was stopped by washing the plate with deionized water, and the plates were dried in the dark. Spots were counted using an ELISPOT reader system (Cellular Technology Ltd.), and the results were expressed as the mean number of specific IFN-γ spot-forming cells per 1 × 10^6^ splenocytes. The procedure for the detection of IL-2, IL-4 and IL-10 were similar to IFN-γ.

### Challenge with HTNV and detection of HTVN-specific antigens in livers and spleens of mice

Based on previous work in our laboratory [18], ten days after the final booster immunization, mice were injected intramuscularly (caudal thigh) with 1×10^5^ FFU (focus forming units) of HTNV. 3 days after HTNV inoculation, mice were sacrificed. The spleens and livers were removed, ground in PBS, lysed by three consecutive freeze-thaw cycles and precipitated by centrifugation. HTNV antigen in the supernatant was detected by the sandwich ELISA previously described [20].

### Statistical analysis

Statistical analysis was performed using Graphpad software version 5.0. One-way ANOVA was used to determine statistically significant differences among the experimental groups. Student's t-tests were used to determine significant differences between experimental and control groups. P-values of 0.05 or less were considered to be statistically significant.

## Competing interests

The authors declare that they have no competing interests.

## Authors’ contribution

YL designed the study, carried out all experiments, performed the statistical analysis and drafted the manuscript. BWT and WXA participated in the design of the study, carried out the experiments and helped to draft the manuscript. ZL, ZL, LPY, WF and LZY helped to carry out part of the experiments. ZFL and XZK conceived of the study, and participated in its design and coordination and helped to draft the manuscript. All authors read and approved the final manuscript.

## Authors’ information

Lan Yu, Wentao Bai and Xingan Wu are co-first authors.
